# Awareness Among Parents About Cotton Earbud Use in Children in the Western Region, Saudi Arabia: A Cross-Sectional Study

**DOI:** 10.7759/cureus.91891

**Published:** 2025-09-09

**Authors:** Muhanna A Alhusayni, Alaa Othman, Salam A Sait, Ghadi S Aljohani, Nouf A Alqarni, Abrar A Alzahrani, Abdulrahman R Albogami, Abdulsalam M Alamri, Manar B Almanbahi, Azza A Taha, Sarah H Abuduruk

**Affiliations:** 1 College of Medicine, Taif University, Taif, SAU; 2 Otolaryngology - Head and Neck Surgery, King Abdullah Medical City, Jeddah, SAU; 3 Otolaryngology - Head and Neck Surgery, King Abdullah Medical City, Makkah, SAU; 4 College of Medicine, Bisha University, Bisha, SAU; 5 Family and Community Medicine, Taif University, Taif, SAU; 6 Otolaryngology, Alhada Armed Forces Hospital, Taif, SAU

**Keywords:** awareness, children, cotton swabs, ear hygiene, ear infections, earwax buildup, parents, saudi arabia

## Abstract

Background and objectives

The use of cotton swabs for ear cleaning is a widespread practice among parents, often perceived as a convenient and effective method to remove earwax. However, there is increasing concern regarding the health risks associated with cotton swab use, particularly in children. These risks include ear canal damage, infections, earwax blockage, and even eardrum perforation. This study aims to assess the prevalence of cotton swab use among parents in the Western Region of Saudi Arabia, evaluate their awareness of the potential health risks, and explore the factors influencing ear hygiene practices for children.

Methods

This cross-sectional study was conducted between July and August 2024, targeting 705 parents residing in the Western Region of Saudi Arabia. Participants were selected randomly, with exclusion criteria that included individuals under the age of 18 and more. A validated, reliability-tested online questionnaire in Arabic was used to collect data. The questionnaire consisted of 25 questions designed to gather demographic information and evaluate parents’ knowledge, attitudes, and practices related to the use of cotton swabs for ear cleaning, as well as their awareness of the associated health risks. Statistical analysis was performed using SPSS version 23.0 (IBM Corp., Armonk, USA). Categorical data were analyzed using chi-square and Fisher’s exact tests, with a significance level set at p < 0.05 for all statistical analyses.

Results

A total of 705 parents participated in the study, of whom 66.2% were female. The majority (29.3%) were aged between 36 and 45 years, and 82% were married. Most participants (72%) had university degrees, and 82.4% worked in non-medical professions. A significant majority (96.7%) were Saudi nationals. Regarding cotton swab use, 28.3% of parents reported using cotton swabs to clean the inside of their children’s ears, with many also using them to clean both the inside and outside of the ear. The most common reason for using cotton swabs was earwax removal (30.2%). Most parents (37.9%) had used cotton swabs for over five years, and 60.4% reported using them less than once a day. Despite the widespread use of cotton swabs, 72% of participants acknowledged the associated risks, including earwax buildup, infections, and eardrum perforation. However, only 58.5% had received prior health education about these risks, with otolaryngologists being the primary source of information. Notably, 13% of parents reported complications related to cotton swab use, with ear pain being the most common (61%).

Conclusion

This study highlights that, despite moderate awareness of the risks, the use of cotton swabs remains prevalent among parents in the Western Region of Saudi Arabia. The findings underscore the need for targeted health education initiatives led by healthcare professionals to promote safer ear hygiene practices. Raising awareness of the risks associated with cotton swabs and advocating for safer alternatives could help reduce reliance on cotton swabs and minimize ear-related complications in children.

## Introduction

The waxy substance secreted in human ears is known as earwax, and it is also known by the medical term "cerumen." It provides a natural barrier and acidic pH that help protect against infection, serves as a defense mechanism against water and insects, and aids in cleansing, hydrating, and protecting the skin of the ear canal [1]. It is believed that self-cleaning the ear interferes with this natural process and may predispose the ear to certain diseases of the ear [2,3]. It has been reported that most individuals consider cerumen as dirt and harmful to the human body [4,5].

In 1923, Leo Gerstenzang created cotton buds. He thought it safer to use a cotton-tipped swab after seeing his wife clean his infant's ears with wads of cotton on toothpicks. "Q-tips Baby Gays" (Q for quality) was the original name of the product, and the brand is still in use today [6]. The purpose of using Q-tips is to clean the ear auricle (external part), relieve itching, and remove any excess water, among other things [7].

Physicians are aware that the external auditory canal (EAC) has an adequate self-cleaning system and that using earbuds can interfere with this mechanism [8,9]. In 1972, reports of cerumen impaction, otitis externa, and tympanic membrane perforation raised the first medical concerns over the usage of cotton buds. The use of cotton buds in the external auditory canal was then discouraged by the manufacturers [8].

Earbuds injure the ear by forcing cerumen further into the EAC, which can lead to wax impaction. Many people have mistakenly used earbuds for cleaning deeper areas in the ear, leading to some serious complications, including infections. In addition, the literature has extensively reported various grave consequences of persistent earbuds application, including acute EAC injury, otitis externa, cotton bud retention, deafness, and ruptured eardrums [10]. These days, they are a common reason to visit an ENT (ear, nose, and throat) clinic due to their extensive use and the many injuries associated with them [7]. Earbuds misuse has been documented as the most common cause of accidental penetrating trauma of the eardrum among children [8].

Complications from cotton buds are a significant public health concern. In reality, using earbuds can seriously harm the ear in addition to being superfluous. The public has always received warnings from otolaryngologists about the dangers of earbuds use. Individuals are still unaware of these realities [11]. The aim of this study was to evaluate parents’ awareness and knowledge regarding the use of cotton swabs (also known as earbuds or cotton buds in some regions) and their potential adverse effects in Taif, Saudi Arabia.

## Materials and methods

Study design

A survey was conducted in this cross-sectional study to assess the awareness among parents about the use of cotton earbuds in children in the Western Region, Saudi Arabia, from July to August 2024. The survey method was chosen to gather data from a large sample within a short period.

Study population

The target population included parents residing in the Western Region with children aged 0-18 years. The sample size was determined based on the prevalence of ear health issues in children and the desired confidence level.

Sample size calculation

The sample size for this study was calculated using the Raosoft sample size calculator (Raosoft Inc., Seattle, USA). Assuming a population size of approximately 10 million individuals residing in the Western Region of the Kingdom of Saudi Arabia (KSA), and an expected prevalence rate of 50% (to ensure maximum sample size and account for unknown variability), a confidence level of 95%, and a margin of error of 5%, the minimum required sample size was determined to be 385 participants.

Data collection

We used an online validated self-administered Arabic questionnaire adapted from a similar prior study [11]. The questionnaire was distributed to the public of Taif City, Saudi Arabia, via a Google Form link shared on social media platforms. The electronic informed consent was obtained prior to the questions. The form was anonymously filled. The questionnaire included 25 questions collecting data on demographic characteristics of the responders (7 questions), their knowledge about the risks associated with cotton earbud use (8 questions), and parental practices and attitudes (10 questions) toward child ear cleaning.

Data analysis

Data was collected in an Excel sheet (Microsoft Corporation, Redmond, USA) and then transferred into SPSS software, version 23.0 (IBM Corp., Armonk, USA), for statistical analysis. Missing or incomplete responses were excluded. Percentages and frequencies were used to describe the demographics of participants, their practice concerning ear cleaning, their attitude about cleaning their ears, the pros and cons of using cotton earbuds, the occurrence of complications due to the use of earbuds, whether participants had previous health education about risks associated with the use of cotton earbuds, and their source of information. Chi-square tests were used for all variables, except for the relationships between practice and educational level, and attitude and educational level, where Fisher’s Exact Test was applied because at least one cell had an observed count less than 5.

Ethical considerations

Ethical approval was obtained from the Research Ethics Committee of Al-Hada Armed Forces Hospital at Taif, Saudi Arabia (application number: H-02-T-078). All participants agreed to participate in the study after being informed of its objectives. Data confidentiality was maintained during and after the study.

## Results

Participants

A total of 705 people participated in the study. Most participants were females (n = 477, 66.2%), age category 36-45 years (29.3%), and married (82%). Most had university education, were employed, and had a non-medical specialty (72%, 53.5% and 82.4% respectively). About 96% resided in the urban area, 36.6% were from Taif city, and 96.7% were Saudi. Most participants had a monthly income between 5000 and 15000 Saudi Riyal (52.7%). Social and demographic characteristics are shown in Table 1.

**Table 1:** Socio-demographic characters of the participants.

Socio-demographic characters	Number (%)
Gender:	
Male	228 (33.8)
Female	447 (66.2)
Age in years:	
< 25	124 (18.3)
26-35	122 (18.1)
36-45	198 (29.3)
46-55	163 (24.1)
>55	68 (10.1)
Marital status:	
Widow	31 (4.6)
Single	20 (3)
Married	554 (82.1)
Divorced	70 (10.4)
Educational level:	
Postgraduate	56 (8.3)
Primary	13 (1.9)
Secondary	120 (17.8)
University	486 (72)
Occupation:	
Student	84 (12.4)
Unemployed	154 (22.8)
Retired	76 (11.3)
Employed	361 (53.5)
Speciality:	
Non-medical	563 (83.4)
Medical	112 (16.6)
Residence:	
Rural	28 (4.1)
Urban	647 (959)
City:	
Taif	247 (36.6)
Madina	148 (21.9)
Jeddah	100 (14.8)
Mecca	130 (19.3)
Yanboa	50 (7.4)
Nationality:	
Saudi	653 (96.7)
Non-Saudi	22 (3.3)
Income:	
< 5000	170 (25.2)
5000 – 10000	165 (24.4)
10000-15000	191 (28.3)
>15000	149 (22.1)

Earbud use

About 28.3% of participants used cotton earbuds for inside cleaning only, and both outside and inside cleaning. The most common cause for using earbuds was for cleaning ears (30.2%), and the most common tool used was cotton earbuds. Most participants (37.9%) had been using cotton earbuds for more than 5 years, and most (60.4%) used them at a frequency less than once daily. Details of the practices related to earbud use are presented in Table 2.

**Table 2:** Practice of using cotton earbuds among participants.

Practice of using cotton earbuds	Number (%)
Do you use cotton earbuds for cleaning children's ears:	
No	208 (30.8)
For outside cleaning only	276 (40.9)
For inside cleaning only	39 (5.8)
For both sides cleaning	152 (22.5)
Reason for cleaning child's ears:	
Not used	208 (30.8)
To remove earwax	115 (17)
To relieve irritation or itching	15 (2.3)
To relieve blockage	4 (0.6)
As a habit	5 (0.7)
For cleaning	204 (30.2)
After bath or swimming to remove water	124 (18.4)
Tools used for cleaning:	
None	208 (30.9)
Cotton earbuds only	300 (44.4)
Cotton earbuds besides other tools (key, pencil, tissue, eardrops, olive oil)	167 (24.7)
Duration of use of cotton earbuds:	
None	208 (30.9)
<5 years	211 (31.2)
>5 years	256 (37.9)
Frequency of use of cotton earbuds:	
None	208 (30.9)
Sometimes	408 (60.4)
Daily	40 (5.9)
Twice daily	11 (1.6)
>Twice daily	8 (1.2)
None	208 (30.9)

Attitude towards earbud use and ear cleaning

About 37% of the participants reported that cotton earbuds were useful in removing earwax, and 29% thought that cotton earbuds were useful and safe. On the other hand, participants reported cotton earbuds can cause ear infection, perforation of the drum, and further earwax impact - 35%, 68%, and 72%, respectively (Table 3).

**Table 3:** Attitude about pros and cons of using cotton earbuds among participants.

Attitude about pros and cons of using cotton earbuds	Responses (Number (%))		
	Yes	No	Not sure
Cotton earbuds effective in removing ear wax	251 (37.2)	166 (24.6)	258 (38.2)
Cotton earbuds are useful and safe	194 (28.7)	195 (28.9)	286 (42.4)
Cotton earbuds can cause ear infection	238 (35.3)	101 (15)	336 (49.8)
Cotton earbuds can cause perforation of the drum	460 (68.1)	43 (6.4)	172 (25.5)
Cotton earbuds can cause further earwax impaction	486 (72)	26 (3.9)	163 (24.1)

With regard to participants’ attitudes towards cleaning ears, responses are presented in Table 4. Participants agreed on cleaning ears by either using cotton earbuds, a wet towel, or a piece of cloth, or by using water only in 35.7%, 68%, and 40% of responses, respectively. However, only 16.7% of the participants agreed that it is better not to clean ears.

**Table 4:** Attitude about cleaning ears among participants:

Attitude about cleaning ears	Responses (Number (%))				
	Strongly agree	Agree	Neutral	Disagree	Strongly disagree
We should use cotton earbuds for cleaning ears	42 (6.2)	199 (29.5)	216 (32)	157 (23.3)	61 (9)
We should use wet towel or piece of cloth for cleaning ears	95 (14.1)	364 (53.9)	142 (21)	56 (8.3)	18 (2.7)
We should only use water for cleaning ears	53 (7.9)	216 (32)	222 (32.9)	150 (22.2)	34 (5)
It is better not to clean ears	21 (3.1)	92 (13.6)	131 (19.4)	278 (41.2)	153 (22.7)

Awareness about earbud use

**Figure 1:**
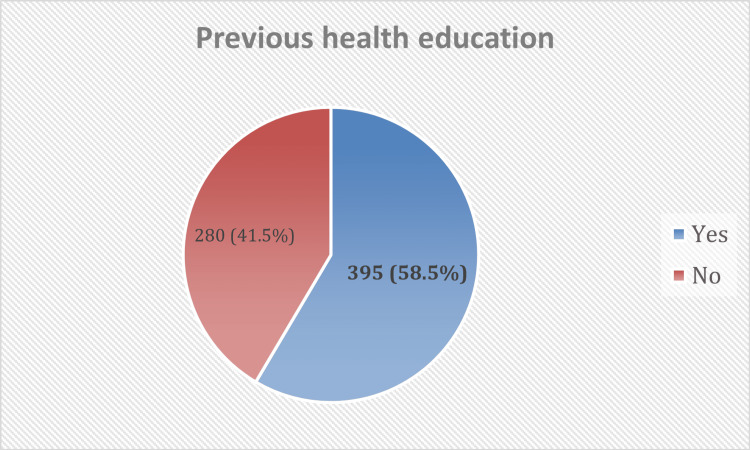
Previous health education about risks associated with the use of cotton earbuds among participants.

**Figure 2:**
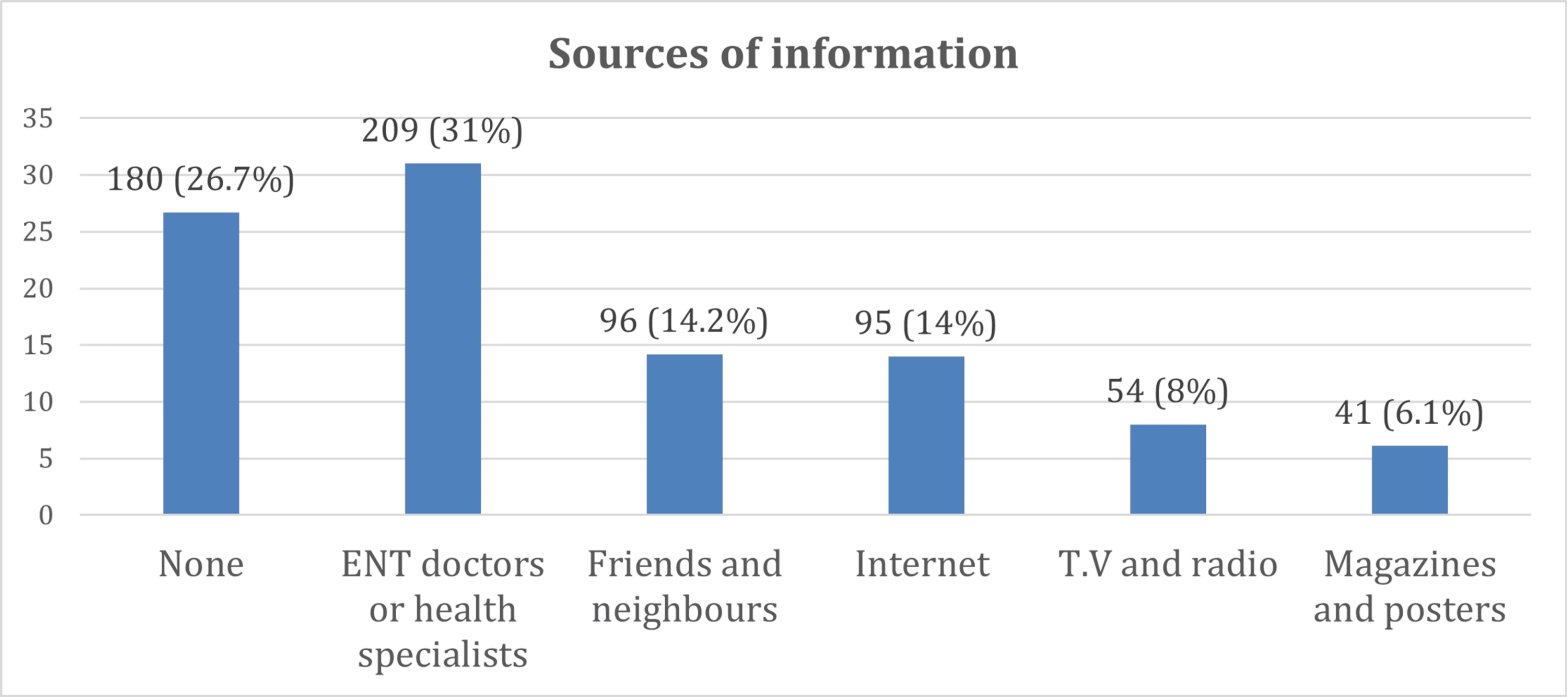
Sources of information about cotton earbuds among participants.

Table 2 shows that about 28.3% of participants used cotton earbuds for both inside cleaning only and both side cleaning. The most common cause for using earbuds was for achieving cleaning of ears (30.2%), and the most common tool used was cotton earbuds. Most participants (37.9%) had been using cotton earbuds for more than 5 years, and most of them (60.4%) used them at a frequency less than once daily. Participants (58.5%) had previous health education about risks associated with the use of cotton earbuds. Most got their information from ENT doctors or health specialists (31%) (Figures 1, 2).

Experience of complications

**Figure 3:**
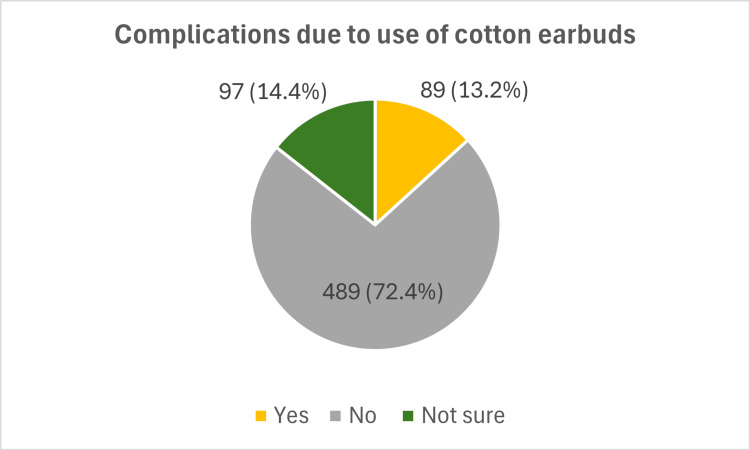
Complications due to the use of cotton earbuds among participants.

**Figure 4:**
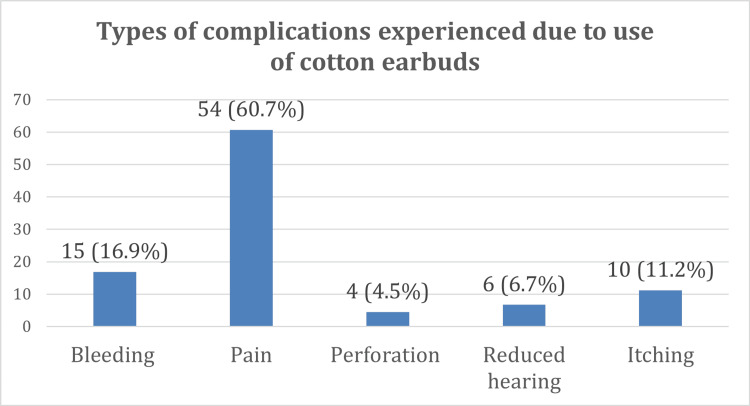
Types of complications experienced due to use of cotton earbuds among participants.

Only 13% of the participants experienced complications due to the use of cotton earbuds. The most common complication noted was pain in 61% of the participants, as shown in Figures 3, 4.

Tables 5, 6 present the association between participants' demographic characteristics and their ear-cleaning practices, as well as their attitudes towards using earbuds for ear cleaning. Among the demographic variables, only gender showed a significant association with ear-cleaning practices, with a higher proportion of females (323, 72.3%) engaging in ear cleaning compared to males (144, 63.2%).

**Table 5:** Association between practice of ear cleaning among participants and their demographic characters.

Socio-demographic characters	Practice ear cleaning	No practice	Test statistic	P value
	Number (%)	Number (%)		
Gender:			5.9	0.015
Male	144 (63.2)	84 (36.8)		
Female	323 (72.3)	124 (27.7)		
Age in years:			7.5	0.110
< 25	80 (64.5)	44 (35.5)		
26-35	87 (71.3)	35 (28.7)		
36-45	127 (64.1)	71 (35.9)		
46-55	121 (74.2)	42 (25.8)		
>55	52 (76.5)	16 (23.5)		
Marital status:			0.47	0.925
Widow	22 (71)	9 (29)		
Single	14 (70)	6 (30)		
Married	385 (69.5)	169 (30.5)		
Divorced	46 (65.7)	24 (34.3)		
Educational level:			2.2	0.552
Postgraduate	39 (69.6)	17 (30.4)		
Primary	7 (53.8)	6 (46.2)		
Secondary	87 (72.5)	33 (27.5)		
University	334 (68.7)	152 (31.3)		
Occupation:			3.6	0.304
Student	53 (63.1)	31 (36.9)		
Unemployed	101 (65.6)	53 (34.4)		
Retired	54 (71.1)	22 (28.9)		
Employed	259 (71.7)	102 (28.3)		
Speciality:			2.1	0.146
Non-medical	71 (63.4)	41 (36.6)		
Medical	396 (70.3)	167 (29.7)		
Residence:			1.98	0.159
Rural	16 (57.1)	12 (42.9)		
Urban	451 (69.7)	196 (30.3)		
Nationality:			1.1	0.297
Saudi	454 (69.5)	199 (30.5)		
Non-Saudi	13 (59.1)	9 (40.9)		
Income:			2.2	0.525
< 5000	112 (65.9)	58 (34.1)		
5000 – 10000	121 (73.3)	44 (26.7)		
10000-15000	131 (68.6)	60 (31.4)		
>15000	103 (69.1)	46 (30.9)		

**Table 6:** Association between attitude of ear cleaning among participants and their demographic characters.

Socio-demographic characters	Earbuds should be used for ear cleaning	Earbuds should not be used for ear cleaning	Neutral	Test statistic	P value
	Number (%)	Number (%)	Number (%)		
Gender:				1.4	0.502
Male	80 (35.1)	80 (35.1)	68 (29.8)		
Female	161 (36)	138 (30.9)	148 (33.1)		
Age in years:				4.1	0.848
< 25	47 (37.9)	34 (27.4)	43 (34.7)		
26-35	49 (40.2)	35 (28.7)	38 (31.1)		
36-45	65 (32.8)	71 (35.9)	62 (31.3)		
46-55	57 (35)	55 (33.7)	51 (31.3)		
>55	23 (33.8)	23 (33.8)	22 (32.4)		
Marital status:				9.5	0.149
Widow	13 (41.9)	10 (32.3)	8 (25.8)		
Single	12 (60)	3 (15)	5 (25)		
Married	194 (35)	186 (33.6)	174 (31.4)		
Divorced	22 (31.4)	19 (27.1)	29 (41.4)		
Educational level:				5.6	0.464
Postgraduate	18 (32.1)	14 (25)	24 (42.9)		
Primary	3 (23.1)	4 (30.8)	6 (46.2)		
Secondary	47 (39.2)	36 (30)	37 (30.8)		
University	173 (35.6)	164 (33.7)	149 (30.7)		
Occupation:				10.2	0.115
Student	34 (40.5)	24 (28.6)	26 (31)		
Unemployed	55 (35.7)	54 (35.1)	45 (29.2)		
Retired	21 (27.6)	35 (46.1)	20 (26.3)		
Employed	131 (36.3)	105 (29.1)	125 (34.6)		
Speciality:				1.3	0.518
Non-medical	204 (36.2)	184 (32.7)	175 (31.1)		
Medical	37 (33)	34 (30.4)	41 (36.6)		
Residence:				1.6	0.458
Rural	9 (32.1)	12 (42.9)	7 (25)		
Urban	232 (35.9)	206 (31.8)	209 (32.3)		
Nationality:				0.278	0.870
Saudi	233 (35.7)	210 (32.2)	210 (32.2)		
Non-Saudi	8 (36.4)	8 (36.4)	6 (27.3)		
Income:				8.2	0.229
< 5000	66 (38.8)	50 (29.4)	54 (31.8)		
5000 – 10000	67 (40.6)	53 (32.1)	45 (27.3)		
10000-15000	55 (28.8)	71 (37.2)	65 (34)		
>15000	53 (35.6)	44 (29.5)	52 (34.9)		

## Discussion

This study investigated the practices, attitudes, and public awareness related to cotton earbud use for ear cleaning in the Western Region of Saudi Arabia. The demographic profile of the study participants was primarily female (66.2%), aged between 36 and 45 years (29.3%), and predominantly married (82%). In addition, most participants were urban residents (95.9%), which may result in better access to health information and practices in comparison to rural areas.

Ear-cleaning practices

A notable finding from this study was the widespread use of cotton earbuds for cleaning the ear canal or both the canal and the outside of the ears (28.3%). This is consistent with findings from other local studies in Saudi Arabia as well as other parts of the world [11-13]. This was true even for health practitioners in Saudi Arabia [14]. The prevalence of this practice reflects the people’s beliefs around ear hygiene generally, where there is a common misconception that earwax is unclean and needs to be removed regularly.

Attitudes towards cotton earbuds

The current study found that 37% of participants perceived cotton earbuds as effective for removing earwax. However, 68% acknowledged the risk of eardrum perforation, 72% were aware they can cause further earwax impaction, and 35.3% were aware they can cause ear infections. A study by Shawish et al. also reported contradicting attitudes, where 53% of participants were aware of the potential dangers of earbud use, such as ear pain and otitis externa, but the majority (92%) were still using earbuds of ear hygiene [11]. This highlights the persistent misconceptions about ear hygiene and the knowledge-practice gap, where awareness does not translate into safer practices.

Health education

The finding that 58.5% of participants had prior health education on the risks of cotton earbuds. ENT doctors were the most common source of information (31%), which aligns with local trends showing that specialist advice plays a crucial role in health-related behavior [11]. However, the continued reliance on informal sources such as friends (14.2%) and the internet (14%) suggests a need for more structured public health campaigns in Saudi Arabia to ensure accurate and consistent messages.

Complications from earbud use

In this study, 13% of participants reported experiencing complications, primarily pain (60.7%) and bleeding (16.8%). A study by Amutta et al. reported a complication rate of 25%, whereas Lee et al. reported an injury rate of only 2% [15, 16]. Although we did not include which ear was most affected, Adedeji et al. found that the right ear was at a higher risk of injury due to the right-handedness of most of the population [17].

Recommendations for safe practices

There was a general awareness among participants about safer ear cleaning alternatives, with 68% endorsing the use of a wet cloth and 40% recommending water-only cleaning. Professional organizations like the American Academy of Otolaryngology recommend against introducing any foreign object, including cotton swabs, inside the ear canal, advocating instead for letting the ears self-clean or using other safer external methods [18]. Given the cultural context in Saudi Arabia, public health strategies should consider incorporating targeted education campaigns to provide evidence-based recommendations for safer alternatives.

Limitations

Limitations of the study include self-reported data, which may be subject to recall bias, and a predominantly urban sample, potentially limiting the generalizability of the findings to rural areas. Future research should aim to include a more diverse demographic population, explore the underlying factors influencing the knowledge-practice gap, and evaluate the effectiveness of different educational interventions in promoting safer ear hygiene behaviors.

## Conclusions

The persistence of cotton earbud use, even among individuals who are aware of the potential risks, underscores the intricate challenges involved in altering entrenched health behaviors within large populations. This phenomenon is not merely a matter of knowledge deficit, but rather a complex interplay of cultural habits, perceived convenience, and misinformation. The findings from this study - reinforced by a growing body of similar research - highlight the urgent need for comprehensive and sustained public health strategies aimed at addressing the widespread and often habitual use of cotton earbuds. To effectively bridge the gap between awareness and safe health practices, public health interventions must go beyond traditional educational campaigns. Multifaceted approaches are essential, incorporating evidence-based guidance from medical professionals, structured health education in schools, and the strategic use of social media to influence public attitudes and norms. Social media platforms, in particular, offer a powerful avenue for disseminating accurate information and countering harmful myths through relatable content, testimonials, and influencer-driven messaging.
